# The Effect of Heterogeneity on Invasion in Spatial Epidemics: From Theory to Experimental Evidence in a Model System

**DOI:** 10.1371/journal.pcbi.1002174

**Published:** 2011-09-29

**Authors:** Franco M. Neri, Anne Bates, Winnie S. Füchtbauer, Francisco J. Pérez-Reche, Sergei N. Taraskin, Wilfred Otten, Douglas J. Bailey, Christopher A. Gilligan

**Affiliations:** 1 Department of Plant Sciences, University of Cambridge, Cambridge, United Kingdom; 2 Department of Molecular Biology, Aarhus University, Aarhus, Denmark; 3 Department of Chemistry, University of Cambridge, Cambridge, United Kingdom; 4 St. Catharine's College and Department of Chemistry, University of Cambridge, Cambridge, United Kingdom; 5 The SIMBIOS Centre, University of Abertay Dundee, Dundee, United Kingdom; 6 INRA UMR1099 BiO3P (Biology of Organisms and Populations Applied to Plant Protection), Le Rheu, France; University of New South Wales, Australia

## Abstract

Heterogeneity in host populations is an important factor affecting the ability of a pathogen to invade, yet the quantitative investigation of its effects on epidemic spread is still an open problem. In this paper, we test recent theoretical results, which extend the established “percolation paradigm” to the spread of a pathogen in discrete heterogeneous host populations. In particular, we test the hypothesis that the probability of epidemic invasion decreases when host heterogeneity is increased. We use replicated experimental microcosms, in which the ubiquitous pathogenic fungus *Rhizoctonia solani* grows through a population of discrete nutrient sites on a lattice, with nutrient sites representing hosts. The degree of host heterogeneity within different populations is adjusted by changing the proportion and the nutrient concentration of nutrient sites. The experimental data are analysed via Bayesian inference methods, estimating pathogen transmission parameters for each individual population. We find a significant, negative correlation between heterogeneity and the probability of pathogen invasion, thereby validating the theory. The value of the correlation is also in remarkably good agreement with the theoretical predictions. We briefly discuss how our results can be exploited in the design and implementation of disease control strategies.

## Author Summary

Pathogen spread and epidemic invasion in plant, animal and human populations depend on host properties (infectivity, susceptibility) that can vary amongst hosts within the same population. However, such host variability (or heterogeneity) is typically difficult to control experimentally, and little explicit research has been done on its effects on pathogen invasion. We present the first systematic investigation on the spread of a pathogen (the fungal plant pathogen *Rhizoctonia solani*) in repeatable microcosm populations (nutrient sites, representing hosts, on a lattice) with varying heterogeneity. Recent theoretical models for heterogeneity make the following prediction, that we set as our hypothesis: increasing heterogeneity (keeping all other properties fixed) results in a decrease of the probability of pathogen invasion. We validate the hypothesis using a combination of statistical methods. Moreover, we find that the decrease in probability of invasion is also in good quantitative agreement with the theoretical predictions. We discuss how our results can be exploited for disease control strategies that increase the heterogeneity of a system in order to limit pathogen spread.

## Introduction

Host heterogeneity is receiving increasing attention as one of the factors affecting the dynamics of epidemic spread. The properties of individual hosts, such as contact rate, susceptibility, or infectiousness, can vary across a population as a result of environmental [1], [2], genetic [3] and immunogenetic [4] factors. Such variability is typically difficult to measure empirically, and has been successfully quantified only in a few significant cases, concerning plant [5], animal [6]–[10], and human diseases [6], [7], [11]. Even more important, a few studies [6], [8]–[10], [12] succeeded in addressing a key epidemiological question: what is, if any, the effect of individual variability on the risk of *epidemic invasion* [13] (that is, the chance that a pathogen, starting from a single or a few infected hosts, will be able to infect a significant proportion of the whole population). For example, it was found that variations in prevalence of *E. coli* O157 among cattle populations were best explained by individual variability in bacterial load and infectiousness[8]; in plant populations, the rate and pattern of disease invasion were found to be influenced by variations in individual susceptibility and transmission rates [2], [14]. An important consequence of these findings is that variability can affect *invasion thresholds*, i.e., the critical values of the parameters of the system (transmission rate, host density, etc.) that determine whether or not a pathogen can invade [13], [15]. Since invasion thresholds are a central idea underlying most control strategies [13], the practical implications are huge: it is known that control strategies can benefit from the knowledge of host variability [11], [12]; on the other hand, such strategies can fail if variability is ignored and “averaged out” [6], [7], [12], due to serious misestimation of the parameters of the epidemic model [14]. However, despite such implications, and a continuing effort to explore the problem with theoretical models (e.g., for fully mixed populations [16]–[20], metapopulations models [21], [22], and complex networks [23]–[25]), rigorous experimental testing has been limited, restricting our understanding of the problem.

The experimental results presented here test for the first time the existence of a link between host heterogeneity and epidemic thresholds in a broad, relevant class of spatially-extended systems, thereby confirming recent theoretical predictions [26]. The class comprises those systems where the pathogen is transmitted between neighbouring hosts: such mode of transmission is typical of many diseases, such as soil-borne diseases in plant populations [27]–[29]; plant pathogens spreading among neighbouring fields or farms [30]; animal pathogens spreading within populations of hosts living in a fixed habitat [31], [32]. A “percolation framework” [33], commonly used to describe these systems, is adopted here to model epidemic spread and invasion. Percolation theory provides conceptual tools that allow “scaling up” from pathogen transmission at the small (between-host) scale to epidemic invasion at the large (population) scale [27]. The use of this framework for the identification of invasion thresholds has been experimentally validated in a few remarkable cases [27], [28], [31], [32]. Many diseases characterized by short-range transmission are also well described by an SIR (susceptible–infected–removed) stochastic model, where an infected host (I) can transmit the pathogen to its susceptible (S) neighbours for some interval of time, after which it is permanently recovered or removed (R); the probability that transmission actually occurs before removal is the *transmissibility*.

Previous experiments [27], [28], using replicable microcosm lattice systems as models for SIR soil-borne plant diseases, succeeded in validating two key predictions from percolation theory [34]: that invasion and threshold behaviour for SIR diseases are controlled by the transmissibility; and that the epidemic threshold value of the transmissibility coincides within experimental precision with the “bond-percolation” threshold for the system ([33]; see Text S1 for details). Hence, a pathogen spreading on a lattice will never invade the population when the transmissibility is lower than the bond percolation threshold, while there will be a risk of invasion when the transmissibility is higher [27], [34].

**Figure 1:**
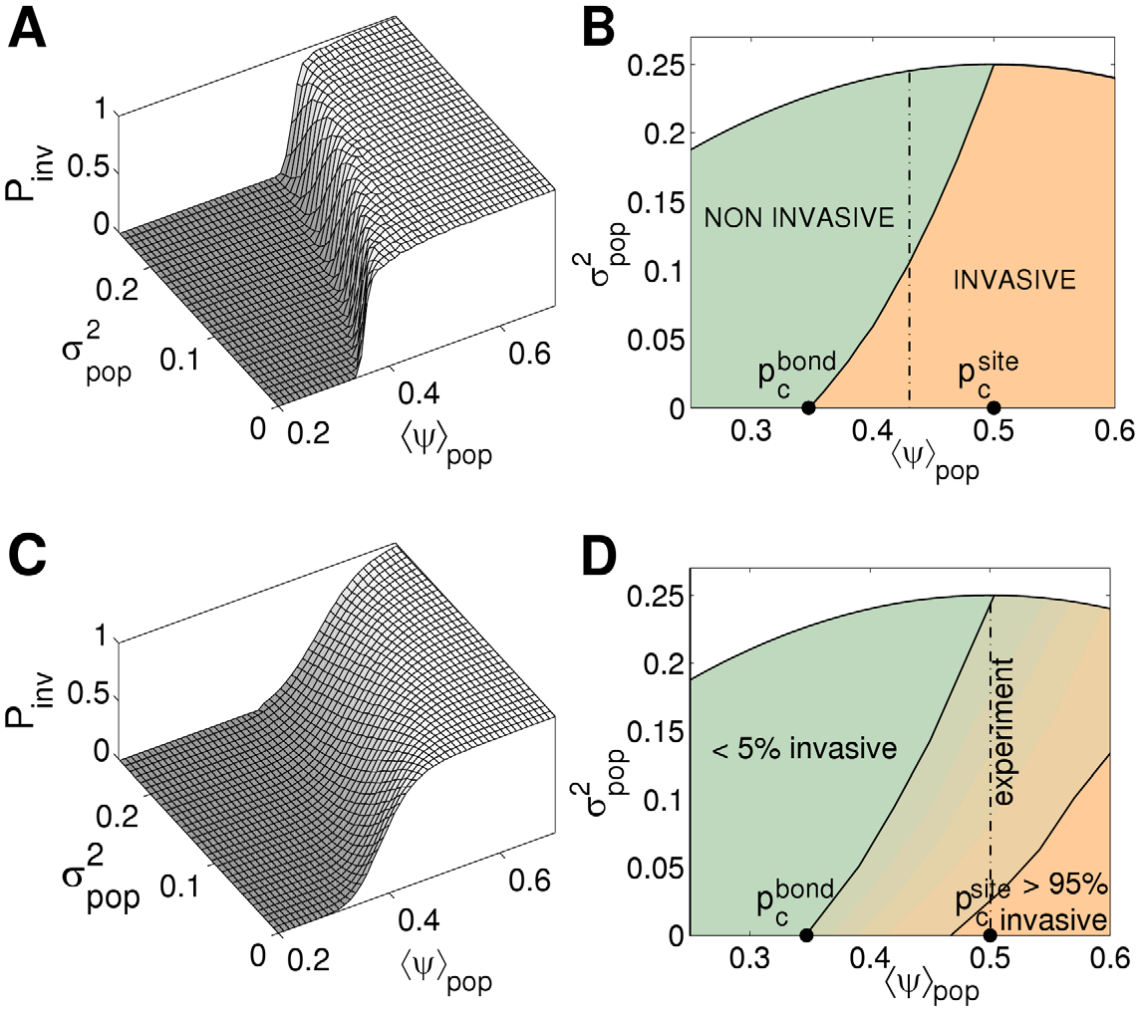
Epidemic invasion in heterogeneous populations: invasion probability and phase diagram. We consider systems of two different sizes, on triangular lattices with the same topology as for the populations used in the experiment. The graphs were obtained with numerical simulations (see Text S1 for details). The probability of epidemic invasion, 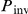, is studied as a function of the mean, 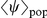, and the variance, 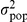, of the transmissibility over the system. (A) Probability of invasion for the larger system (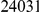 sites) as a function of the mean and variance of the transmissibility. (B) Phase diagram for invasion, calculated numerically from (A). The solid line marks the phase boundary where the transition between the invasive and the non-invasive regimes occurs (corresponding to the sharp jump in (A)). The quantities 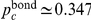 and 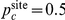 are the bond- and site-percolation thresholds, respectively, for the triangular lattice. The white region beyond the parabolic thick curve corresponds to combinations of values of 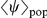 and 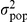 that are theoretically impossible. The large-size phase boundary is shown here to exemplify the (more rigorously defined) phase boundary for infinite-size systems (see discussion in Text S1), for which it provides a good numerical approximation. (C) 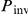 for the smaller system (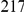 sites). (D) Effective phase diagram for invasion in the small-size system calculated from (C): as expected for such systems, the transition between the two regimes is “smeared out” across a broader region (delimited by solid lines corresponding to the values 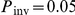 and 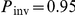). For systems of both sizes, it is possible to change invasion regime by changing the variance 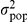 only, with the average 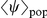 kept constant (dash-dotted lines in (B) and (D); the position of the line for the two systems is different owing to finite-size effects).

The experiment described in the present paper is inspired by a model by Neri *et al.* [26] that goes beyond the percolation-based theory of SIR epidemics by including host variability in the probability of transmission. In a homogeneous system, all the hosts (once infected) are able to transmit the pathogen with the same probability. Conversely, a heterogeneous system, typical of most natural host populations, can be modelled by assuming that the transmissibility is not constant across the population, but is a random variable, drawn for each host from a given common distribution [26]. Epidemic invasion in such systems can then be characterized by two parameters: the average value of the transmissibility over the population and its variance. The variance is used as a measure of the heterogeneity of the system. It was found [26] that both the average and variance of the transmissibility contribute to invasion, but with opposite effects: while the former increases the probability of invasion, the latter leads to a decrease (Figure 1A). As a consequence, invasion can be described by a phase diagram in the two-dimensional parameter space for the average and variance of the transmissibility (Figure 1B). The phase diagram contains two distinct regions: a region with low average transmissibility and large variance, where an epidemic will never invade the system (non-invasive regime), and a region with large average transmissibility and low variance, where the epidemic can invade (invasive regime). Instead of a single threshold value for the transmisibility, a threshold curve (phase boundary) separates the two regimes. A further important result is that, in a given region of the phase diagram (Figure 1B), it appears to be possible to “switch” a system from being invasive to being non-invasive (or *vice-versa*) by keeping the average transmissibility constant and changing only the variance, i.e., by changing only the heterogeneity of the system. The latter result is the main motivation for the present experiment.

We use replicable microcosms [27], [28] to test the predictions of the heterogeneous SIR model[26]. We take advantage of an experimentally validated paradigm [27], [28], whereby the spread of an SIR epidemic in a discrete host population is equivalent to, and can be investigated by, the spread of fungi by mycelial growth among a population of nutrient sites. We analyse the growth of the ubiquitous pathogenic fungus *Rhizoctonia solani* in microcosm populations composed of nutrient sites arranged on a lattice. Here, the term “transmissibility” (which in this particular case is related to infectiousness of donor sites, see Text S1) represents the probability of fungal spread from one site to another. Our main aim is to answer the following questions: does host heterogeneity (measured by the variance of the transmissibility) affect the probability of invasion, and if this is the case, how? We also ask: is it possible to see an effect of heterogeneity on the threshold for invasion of the system? Since our experiments are conducted on relatively small populations, while tresholds are rigorously defined only for infinite systems (Figure 1), we also address the question: can thresholds for invasion be estimated from a small-scale experiment?

We set up a series of notional experimental treatments (replicated populations), designed in such a way to ensure an appropriate range for the average and variance of the transmissibility. The notional values of the parameters are chosen according to the theoretical predictions of Neri *et al.* [26]: the average transmissibility is the same for all the populations, while the variance differs amongst treatments (cf. the dash-dotted line in Figure 1D). This design allows us to determine how the probability of invasion, calculated using spatio-temporal experimental maps, changes with the variance. In practice, because of inherent variability of the systems, replicates within the same treatment differ. Accordingly, at the end of the experiment, we re-estimate the values of the average and variance of the transmissibility for *each* individual replicate from spatio-temporal maps, using Bayesian Markov-chain Monte Carlo (MCMC) methods [35]. The new estimated parameters are then used for a statistical analysis carried out on the pooled set of all the populations. The pooled analysis proves to be effective in assessing the joint contribution of average and variance of the transmissibility to the probability of invasion.

## Results

### Invasive spread and individual rates in heterogeneous populations

Six experimental treatments, labelled from A to F, were designed (see below and Table S1), with 30 replicated populations for each treatment. Each treatment corresponded to a population of 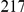 nutrient sites (agar dots) arranged on a triangular lattice, comprising a fraction, 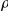, of “occupied” sites (randomly selected to be occupied with nutrient), the remaining fraction 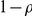 being left empty. Henceforth, the symbol 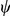 will used to denote transmissibility in general; 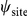 for the transmissibility of a site with nutrient; 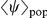 and 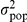 for the mean and variance, respectively, of the transmissibility over a population. The value of 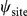 depends on the amount of nutrient (see Text S2 for the determination of 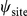). The population mean and variance of the transmissibility of the experimental populations are given by:
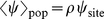
(1a)

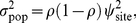
(1b)and were controlled by adjusting 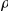 and 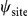 in order to keep 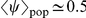 approximately constant for all treatments (cf. the dash-dotted line in Figure 1D), while 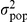 was increased by regular intervals, in alphabetical order, from A (homogeneous system) to F (maximally heterogeneous system; see Text S1 for more details).

**Figure 2:**
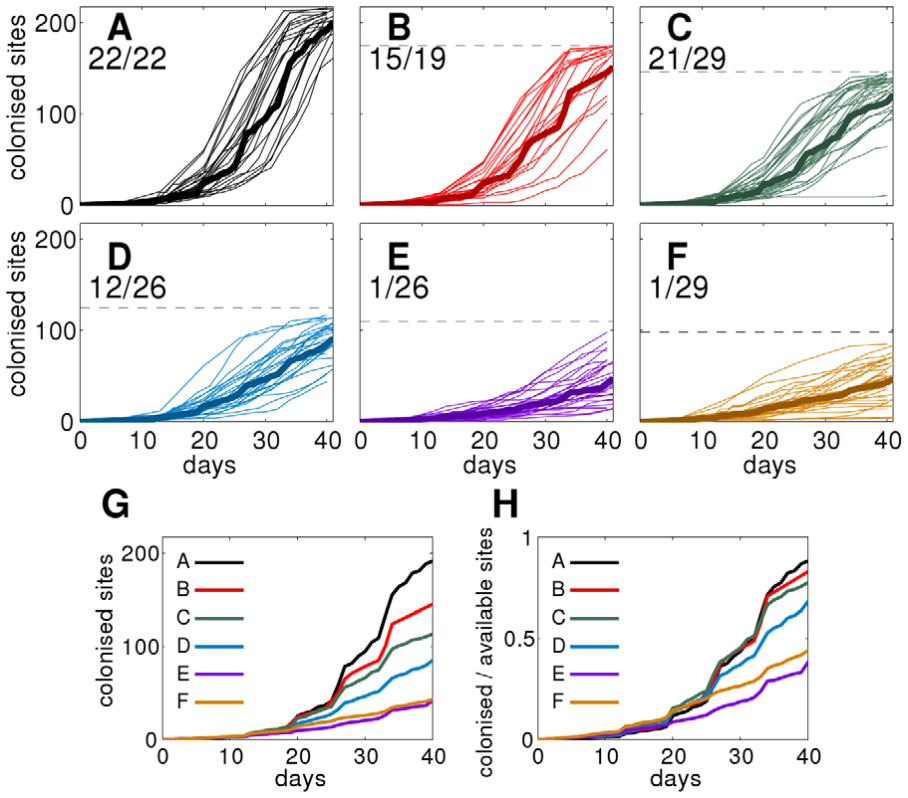
Experimental colonisation curves for the six different treatments. (A–F) Individual colonisation curves for each of the replicates of a treatment (thin lines) and the average over all the replicates (bold solid line). The upper limit of the vertical axis in all the panels concides with the total number of sites in the population (i.e., 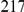), while the horizontal dashed line in treatments B to F marks the number of available sites (i.e., occupied by nutrient). The fraction of invading replicates per treatment is displayed in each panel as “number of invading replicates divided by total number of replicates”. (G) Comparison of average colonisation curves for all the treatments. (H) Comparison of average colonisation curves, normalised to the total number of occupied sites per treatment.

Spatio-temporal maps of fungal colonisation dynamics were used, in order to count the cumulative number of colonised sites over time, and to identify those replicates in which the fungus spreads invasively (Figure 2). For each treatment, a fraction of the replicates had to be discarded because of contamination from external sources, leaving a total of 151 replicated populations out of the initial 180. The fraction of invading replicates (Figure 2) shows that the probability of invasion (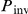) decreased from treatments A to F, following the predicted trend (Table S1). Going from A to F, the fractions of available sites (i.e., occupied by nutrient) reached by the colony also decreased (with the exception of treatments E and F, probably due to stochasticity).

The variability in the final number of colonised sites amongst replicates of the same treatment (Figure 2) can be attributed to two distinct factors: (1) it was partly an effect of the stochastic nature of the colonisation process, which is taken into account by our model, but (2) it could also be caused by variations in the value of 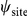 amongst different replicates, due to uncontrollable factors such as variations in the environmental conditions amongst replicate populations contained within different Petri plates. Within-treatment variation of 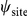, which is not accounted for in our model, can also significantly change the probability of fungal invasion. For this reason, re-assessment of 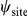 was conducted for each individual replicate in order to test within-treatment variation. In what follows, we call 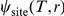 the transmissibility for replicate 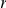 of treatment 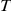 (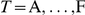), and 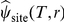 the corresponding estimate (see Materials and Methods for definitions and an explicit example).

In order to estimate 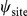, we modelled the time evolution of the probability of transmission between nutrient sites with a Weibull function (see Materials and Methods for details), initially characterized by a single rate of spread. However, preliminary inspection of the population data showed *two* distinct stages for the fungal colony spread: an initially slower process (first stage), followed by a transition to a faster process (second stage). This behaviour can be explained as an effect of nutrient translocation, common to several fungal species [36], [37]: mycelial colonies growing from different nutrient sites are able to share resources, so that their rate of spread increases with the number and connectivity of colonised sites in the system. We accounted for this effect by using a modified Weibull model: the new model included two distinct rates, corresponding to the two stages of the process, and the “switching” time of the transition from the slower to the faster stage. We found that the two-rate model could parametrise the data very efficiently, and provided a good estimation of the posterior distribution for 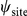 (see Figures S2–4 in Text S3).

**Figure 3:**
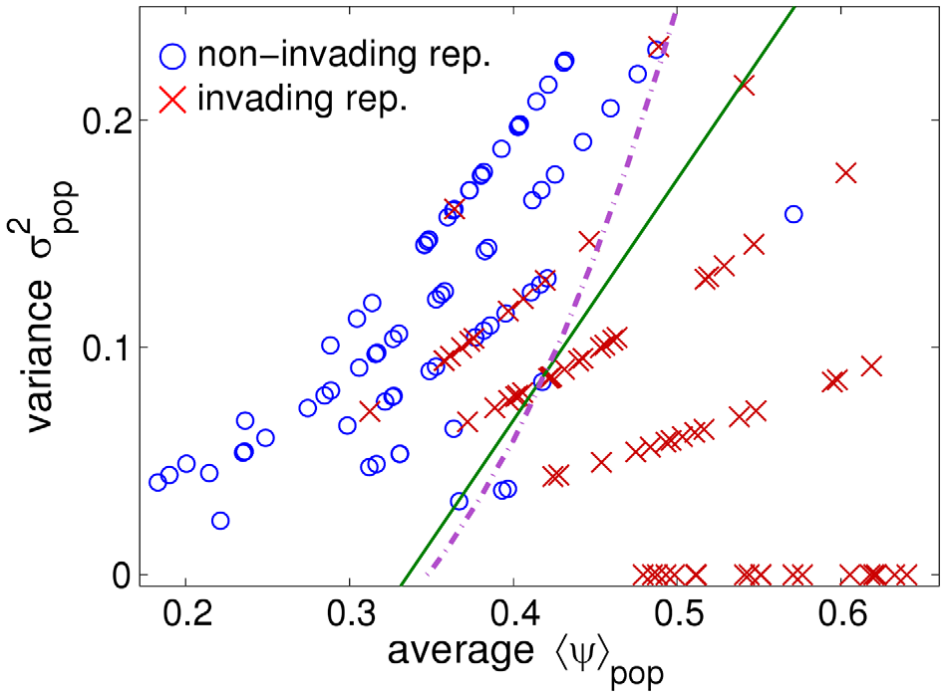
Estimated population parameters and linear discriminant analysis for invasion in the plane 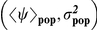. Red crosses and blue circles correspond to invasive and non-invasive replicates, respectively (error bars not shown here). The green thick line is the discriminant function separating the invasive and non-invasive regimes. The purple dash-dotted line is the phase boundary for an infinite system with the same topology (see Text S1), and is close to the solid line for the large-system phase boundary in Figure 1B.

The analysis of posterior distributions for 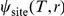 for different replicates, 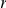, of the same treatment 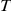, revealed considerable differences from the notional values used for the experimental design (summary statistics in Table S1; see Text S3 for the complete set of results). In particular, 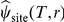 showed a systematic shift to lower values of 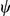 for treatments D, E, F. On average, 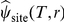 *between* treatments still increased from treatment A to F. However, and most important, within-treatment heterogeneity of 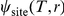 was in general large (Table S1). Such heterogeneity can be modelled explicitly using a hierarchical model [35] (Text S3). For the purpose of the present experiment, however, the main outcome of this analysis is that deviations from the initial, notional values of 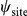 (hence, of 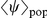 and 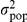) within each treatment are significant. Hence, instead of the nominal values of the parameters, we used the re-estimated values for the final statistical analysis (Figure 3), which was carried out on the pooled set of all the populations. For each population, 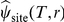 was used to obtain new estimates for the mean and variance of the transmissibility, 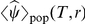 and 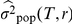 (represented by circles and crosses, respectively, in the 
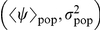
 plane shown in Figure 3).

### Experimental phase diagram for invasion

The data presented in Figure 3 provide evidence for the main hypothesis of the paper: namely, that the variance as well as the mean influence the probability of invasion. Two different statistical tests were used to test the hypothesis. Linear discriminant analysis (LDA) was used to find a linear separatrix between the invasive and non-invasive regimes in the parameter space (solid line in Figure 1C; discriminant function 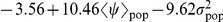). The function classified correctly 75 out of the 79 non-invasive points in the graph (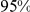 success rate) and 49 out of the 72 invasive points (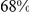 success rate). Goodness-of-fit tests gave Wilks'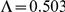, 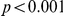. Remarkably, the linear separatrix approximates very well the theoretical prediction for the phase boundary in an infinite system (see Text S1), shown by the dash-dotted line in Figure 3 (and approximated by the solid line for the large system in Figure 1B).

A multiple logistic regression test (function 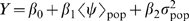, with 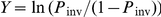) was also conducted, motivated by the fact that the theoretical 3D invasion curves (Figure 1) can be well fitted by a multivariate logistic model. The values of 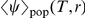 and 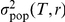 for each population were weighted with the reciprocal of the variance, calculated from the corresponding posterior curves. The test yielded the estimated parameter values (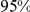 confidence intervals are indicated): 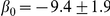, 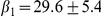, 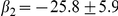 (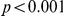 for all the parameters). The coefficient ratio 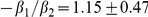 is compatible with the slope 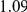 found with LDA. Both tests show the statistical significance of 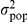 as a predictor for 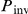.

## Discussion

We have shown experimentally that between-host variability affects the nearest-neighbour spread of a pathogen in a population: when the variability is increased, the probability of epidemic invasion decreases. From a broad point of view, our results answer a very general question: what is the effect of individual variability on disease spread? [6], [7], [38], the answer being valid for systems with a locally spreading pathogen. We have exploited a percolation-based approach, which is widely used for such systems, and which has been experimentally tested for disease invasion in plant [27], [28] and animal populations [31], under the assumption that these populations were homogeneous. Theoretical work [26], [39] has shown how to include heterogeneity in the percolation paradigm, by introducing a quantitative measure of between-host variability [26] (defined as the variance, 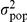, of the transmissibility 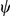 within a population). This way, it becomes possible to make quantitative predictions on pathogen invasion in the presence of heterogeneity.

Here, for the first time, the approach and quantitative predictions of Neri *et al.* [26] have been validated experimentally. We have exploited the saprotrophic spread of *R. solani* , a ubiquitous plant pathogen, in simplified microcosms of hosts represented by agar dots arranged on regular lattices. Previous work showed that such simple microcosm systems are representative of epidemic systems that can be described by SIR spatial models [27], [28]. We have been able to show quantitatively (Figure 3) the effect of within-population heterogeneity (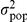) on the probability of epidemic invasion, 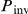, and demonstrated the existence of a statistically significant, negative correlation between 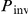 and 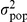. At the same time, our results and analysis show that the phase diagram for invasion (in principle, defined only for infinite systems), which includes the effects of the variance, can be well approximated using small-scale experiments.

Our analysis showed that within-treatment variability can be large enough to mask the effects of experimental treatments in replicated populations (see Table S1 and Text S3). The methodology we adopted is relevant, in general, to the case when experimental factors are subject to environmental stochasticity, thus are not under the full control of the experimenter. Specifically, while one of our experimental factors (the fraction 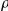 of occupied sites in a population) was known exactly, the other (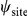) could vary considerably amongst different replicates of the same putative treatment (Figures S2 and S3 in Text S3). Such within-treatment variability is not always necessarily relevant (e.g., in [27], [28]), but it can interfere with the statistical analysis when the values of the experimental parameters need to be known with high precision, as in our case. We showed that within-treatment variability can be efficiently assessed, via MCMC Bayesian techniques, with a *post-hoc* estimation of 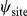 from each individual replicate. The parameter estimation step has also provided an efficient tool to overcome the difficulties due to within-treatment variability. It was indeed possible to analyse the pooled set of the replicates from all the treatments (Figure 3) instead of the average response of a treatment: this approach has proved to be successful in estimating the phase boundary for invasion.

### Variability and implementation of control strategies

**Figure 4:**
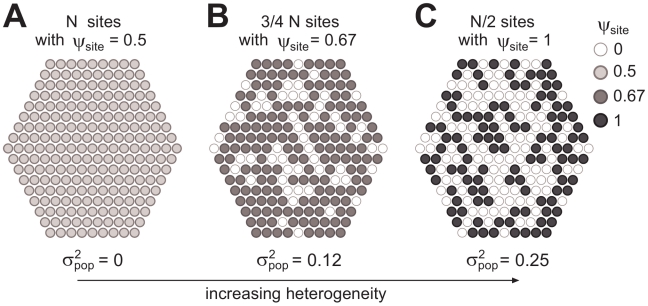
Three experimental treatments for heterogeneous populations of nutrient sites. The treatments presented here are slightly idealized versions of the real experimental treatments A, C, F (the condition 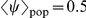 is used here instead of 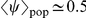). The fraction 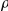 of sites with nutrient and the transmissibility 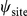 are ajdusted so that 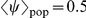 (given by Equation 1) for all the treatments, while heterogeneity (measured by 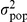 and given by Equation 2) increases. (A) Treatment A: homogeneous system, with all the 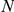 sites occupied (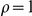) and 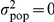. (B) Treatment C, heterogeneous system with 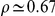 and 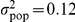. (C) Treatment F: heterogeneous system with 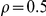 and 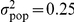 (the “maximally heterogeneous” treatment that maximising the variance given the mean 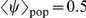).

Our results have a potentially high impact in finding control strategies for the spread of disease. Let us consider a homogeneous system, with the same topology as for our experimental microcosms (Figure 4), but where all the sites have initially a high transmissibility (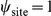, yielding a probability of pathogen invasion 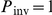). Assume that an epidemic is about to start from the central site, and we can control disease spread by applying, only once, a control agent (a protectant) to all or part of the sites. The effect of the agent on a site is (for simplicity) linear, so that an amount 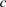 of control agent brings 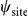 to the value 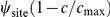, where 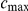 is the amount needed to make the site non-infectious (i.e., 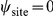). If the amount of control agent at our disposal is fixed and less than 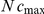 (which would be needed to make all the sites non-infectious), the question is how best to allocate such amount amongst the sites (a similar problem is discussed in [26]).

In particular, if we assume that the amount of control agent is equal to 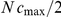, different allocation strategies correspond to our experimental treatments. We could opt for a homogeneous strategy, applying an amount 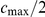 of agent to each individual site, and reducing its transmissibility from 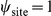 to 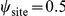 (exemplified by treatment A, Figure 4A). Alternatively, we could apply a heterogeneous strategy: for example, selecting a fraction 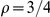 of the sites to be treated with an amount 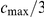 (bringing their transmissibility to 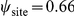), and the remaining fraction (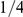) of the sites to be treated with 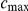 and made non-infectious (exemplified by treatment C, Figure 4C). For any strategy, the value of the final mean transmissibility is always 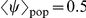, and only 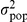 changes. The results presented in this paper show that, if the cost of any strategy is constant (i.e., it depends only on the amount of control agent applied), the maximally heterogeneous strategy (exemplified by treatment F, Figure 4C) gives the maximal decrease of the probability of invasion in every finite system (see [26] for a discussion of analogous results for infinite systems).

## Materials and Methods

### Experimental design

In the experimental design and the subsequent analysis, the parameters were evaluated in two steps, before and after the population experiment. Before the population experiment, the dependence of 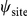 on the agar dot nutrient concentration was estimated by means of “placement” experiments involving only pairs of sites [27], [28] (see Text S2 for the details of the placement experiments and the estimation of 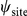). The results of pair experiments were used to select the values of the parameters 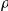 and 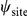 for the notional treatments of the population experiment (Table S1; three of the treatments are exemplified in Figure 4). The real values of 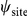, however, can be affected by environmental conditions, and can change significantly amongst replicates of the same treatment. Therefore, after the end of the experiment, new estimates of 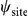 were obtained for each population individually, using Markov-chain Monte Carlo (MCMC) methods, in order to assess within-treatment variability. The new estimates were then used for the pooled analysis shown in Figure 3.

In our experimental systems, each agar dot consisted of a small aliquot (10 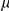
L, 3 mm diameter) of potato dextrose agar (PDA), with nutrient concentrations ranging from 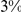 to 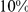. Sites of agar dots were spotted onto a triangular lattice in large Petri plates (140-mm diameter) at 8-mm apart (from centre to centre). Each treatment was replicated 30 times using independent randomisation schemes, leading to a total of 180 populations. The central agar site of each population was inoculated with a single hyphal strand removed from the growing edge of a 4d-old colony of *R. solani* R5 (AG 2-1) grown on water agar. Moist filter paper was placed in the lid of each Petri plate to avoid desiccation of the agar and the plates were sealed and incubated in the dark at 23

C and assessed for 41d using a binocular microscope (40x), recording the number and locations of colonised sites. For each treatment, 20 replicates were assessed every 2 days, and the remaining 10 replicates were assessed weekly. Spatio-temporal maps (snapshots of colonisation over time) were therefore produced.

#### Criteria for invasion

The fact that the transition between the invasive and the non-invasive regime is “smeared out” (Figure 1C) raises the question of how accurately the transition can be approximated in small experimental microcosms. This depends in a crucial way on the criterion used to define invasion. Previous authors [27], [28] considered reaching *at least one edge* of the system boundaries at the end of the experiment (starting from a single infected site at the center) as equivalent to invasion, on the basis that the epidemic is certainly non-invasive when no edge is reached. In this paper, we adopt a different criterion: invasion occurs when *all the edges* are reached by the pathogen at the end of the experiment (i.e., six edges in our case, which is the criterion used in Figure 1). Our choice was made after comparing the probability of invasion for simulated epidemics calculated with the two different criteria (results not shown here). We found that (i) for large system sizes (cf. Figure 1A) the values of 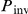 calculated with the two criteria tend to coincide, but (ii) the deviation in probability between large and small systems (Figure 1A versus Figure 1C) is systematically smaller when the “six-edges” criterion is used. Hence, the latter criterion gives a better prediction of the large-scale behaviour of an epidemic from its small-scale behaviour.

### Fungal spread, parameter estimation and data analysis

The value of 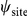 was re-estimated for each individual population at the end of the experiment, using an MCMC method [35]. The growth of the fungal colony between two neighbouring sites was modelled as a time-inhomogeneous Poisson process [40] described by a Weibull distribution multiplied by the transmissibility:
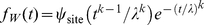
(2a)

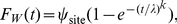
(2b)where 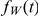 is the distribution of colonisation times, and 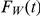 is the probability of colonisation as a function of 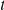; 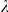 is the time scale of the process and 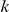 is a shape parameter. In order to account for the observed transition in rates (slower spread at the beginning, faster spread towards the end of the experiment), we introduced a “switching time” 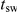, such that 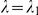 for 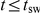 and 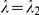 for 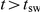 (in general, 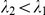). Thus, the model has 5 parameters, represented by the vector 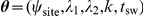. In order to estimate the parameters, we adopted a Bayesian framework, treating the parameters as random variables themselves. The posterior distribution for 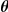 given the observed data 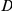, 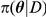, is given by Bayes' formula 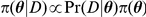, where 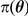 is the prior distribution of the parameters (reflecting our initial belief in their values), and 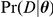 is the likelihood (the probability of the observed data given 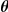). The posterior 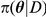 was estimated numerically with the MCMC method and a Metropolis-Hastings algorithm (see e.g. [35]). We refer the reader to Text S3 for details about the algorithm and the estimation process, and for more explicit results.

**Figure 5:**
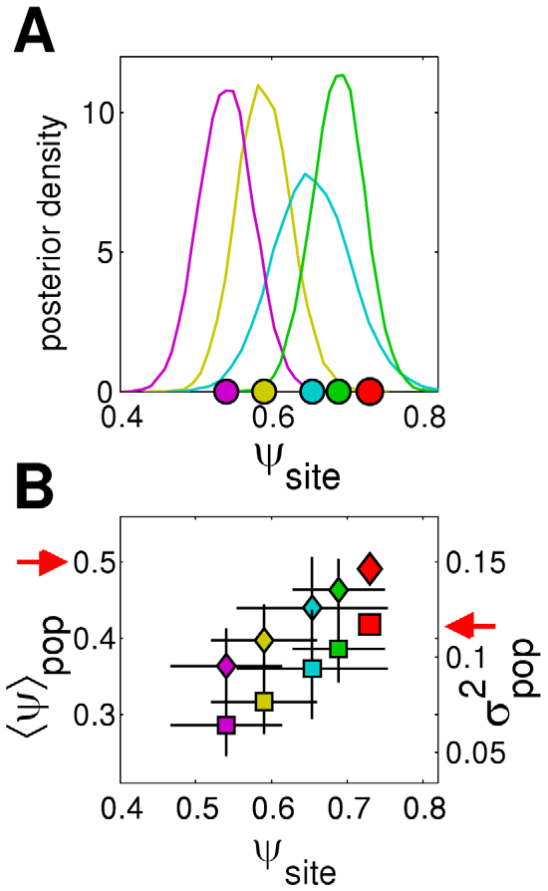
Within-treatment variation of 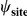, re-estimated with Monte Carlo methods. (A) Posterior distributions for 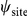 for four different replicates of treatment C (coloured lines). The means of the posteriors (coloured circles) shift away from the value 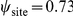 (red circle) used for the experimental design (Table S1). (B) The mean of the posterior is used to re-calculate 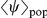 and 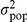 for each replicate (coloured diamonds and squares, respectively), using the 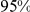 credible interval as the error bar. The new values differ from the nominal values 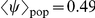 and 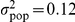 (red diamond and square, respectively, marked by arrows) used in the experimental design (Table S1).

We analysed the posterior distribution for the replicate transmissibility 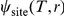 for each replicate 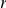 of treatment 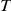. Figure 5A shows as an example the distributions for four different replicates of the same treatment. The mean of the posterior for 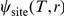 was chosen as the new estimated value of the transmissibility, 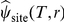 (with error bar corresponding to the 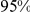 credible interval, Figure 5B). The new estimates for the mean and variance for each population, 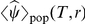 and 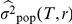 (with the associated confidence intervals), were re-calculated from Equation 0 using 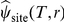, and plotted in the plane 
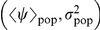
 (Figure 5B). In order to assess the dependence of 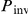 on the estimated values of 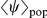 and 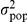, we used multiple logistic regression [41], supplemented by linear discriminant analysis (LDA) [42], to find the line in the plane 
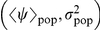
 that best separates the two groups of invasive and non-invasive replicates. All the tests were performed with the R statistical package [43].

## Supporting Information

Table S1
**Notional treatments of the population experiment.** Parameter estimates used for the experimental design are shown here, compared with the corresponding *post-hoc* estimates from the population experiment. The treatments were devised to achieve an approximately constant value of 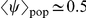, and values of 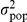 decreasing by approximately regular intervals from 0 to 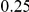. The total number of sites (with and without nutrient) for each population is 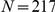. Columns 4 to 7: estimates of 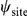, 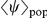, and 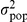 as a function of the nutrient concentration from pair experiments (see Text S2). For each parameter, the best-fit value is indicated in bold face, the 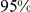 confidence interval is in parentheses. The suffix 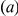 indicates parameters obtained by interpolation between those for 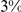 and 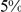 nutrient concentrations. 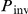 is calculated with numerical simulations (cf. manuscript Figure 1 and see Text S1 for details). Columns 8 to 11: summary statistics for the estimates 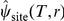, 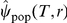 and 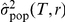, in the form (mean 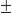 standard deviation); distributions for treatments E and F are significantly asymmetric, see comment in Text S3. The estimate for the probability of invasion, 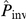, is calculated as the ratio of the number of invading replicates divided by total number of replicates, for each treatment (cf. manuscript Figure 2).(PDF)

Text S1 **We give a theoretical background on percolation theory and epidemic processes in heterogeneous systems.**
(PDF)

Text S2 **We describe the colonisation experiments performed to find the value of the transmissibility as a function of the nutrient concentration.**
(PDF)

Text S3 **We give more details on the MCMC methods used for parameter estimation.** We also provide a complete survey of the results of the estimation, and show how heterogeneity in transmissibility can be described by a simple hierarchical model.
(PDF)
