# Supplementary material for: The Effect of Heterogeneity on Invasion in Spatial Epidemics: From Theory to Experimental Evidence in a Model System
Source: PLoS Comput Biol. 2011 Sep 29;7(9):e1002174. doi: 10.1371/journal.pcbi.1002174 (PMC3182855; doi:10.1371/journal.pcbi.1002174)
Supplement: Table S1 — Notional treatments of the population experiment. Parameter estimates used for the experimental design are shown here, compared with the corresponding post-hoc estimates from the population experiment. The treatments were devised to achieve an approximately constant value of , and values of decreasing by approximately regular intervals from 0 to . The total number of sites (with and without nutrient) for each population is . Columns 4 to 7: estimates of , , and as a function of the nutrient concentration from pair experiments (see Text S2). For each parameter, the best-fit value is indicated in bold face, the confidence interval is in parentheses. The suffix indicates parameters obtained by interpolation between those for and nutrient concentrations. is calculated with numerical simulations (cf. manuscript Figure 1 and see Text S1 for details). Columns 8 to 11: summary statistics for the estimates , and , in the form (mean standard deviation); distributions for treatments E and F are significantly asymmetric, see comment in Text S3. The estimate for the probability of invasion, , is calculated as the ratio of the number of invading replicates divided by total number of replicates, for each treatment (cf. manuscript Figure 2). (PDF) [file pcbi.1002174.s001.pdf]

Table S1: Notional treatments of the population experiment.

Treatment	Nutrient concentration	Number of occupied sites	**Experimental parameters: estimates used to design the experiment**				**Experimental parameters: estimates from populations and within-treatment heterogeneity**			
			$\psi_{\mathbf{site}}$	$\langle\psi\rangle_{\mathbf{pop}}$	$\sigma^{\mathbf{2}}{}_{\mathbf{pop}}$	$\mathbf{P}_{\text{inv}}$	$\hat{\psi}_{\text{site}}(T, r)$	$\hat{\psi}_{\text{pop}}(T, r)$	$\hat{\sigma}^2_{\text{pop}}(T, r)$	$\hat{P}_{\text{inv}}$
**A**	3%	217	**0.51** (0.4, 0.62)	**0.51** (0.4, 0.62)	**0**	**0.98** (0.81, 1)	0.58 ± 0.08	0.58 ± 0.08	0	1
**B**	3.5%	175	**0.62** (0.51, 0.71)$^{(a)}$	**0.5** (0.41, 0.58)$^{(a)}$	**0.06** (0.04, 0.08)$^{(a)}$	**0.91** (0.71, 0.98)	0.62 ± 0.1	0.5 ± 0.08	0.06 ± 0.02	0.79
**C**	4%	146	**0.73** (0.62, 0.81)$^{(a)}$	**0.49** (0.42, 0.54)$^{(a)}$	**0.12** (0.08, 0.14)$^{(a)}$	**0.78** (0.61, 0.90)	0.63 ± 0.12	0.43 ± 0.08	0.09 ± 0.03	0.72
**D**	4.5%	125	**0.84** (0.73, 0.90)$^{(a)}$	**0.48** (0.42, 0.52)$^{(a)}$	**0.17** (0.13, 0.2)$^{(a)}$	**0.67** (0.47, 0.82)	0.66 ± 0.09	0.38 ± 0.05	0.11 ± 0.03	0.46
**E**	5%	110	**0.95** (0.84, 1)	**0.48** (0.43, 0.51)	**0.22** (0.18, 0.25)	**0.14** (0.05, 0.32)	0.7 ± 0.16	0.35 ± 0.08	0.13 ± 0.06	0.04
**F**	10%	98	**1** (0.94, 1)	**0.45** (0.42, 0.45)	**0.25** (0.21, 0.25)	**0.03** (0.01, 0.17)	0.78 ± 0.16	0.35 ± 0.07	0.16 ± 0.05	0.04
