# Supplementary material for: The Effect of Heterogeneity on Invasion in Spatial Epidemics: From Theory to Experimental Evidence in a Model System
Source: PLoS Comput Biol. 2011 Sep 29;7(9):e1002174. doi: 10.1371/journal.pcbi.1002174 (PMC3182855; doi:10.1371/journal.pcbi.1002174)
Supplement: Text S1 — We give a theoretical background on percolation theory and epidemic processes in heterogeneous systems. (PDF) [file pcbi.1002174.s002.pdf]

# The effect of heterogeneity on invasion in spatial epidemics: from theory to experimental evidence in a model system

Franco M. Neri, Anne Bates, Winnie S. Füchtbauer, Francisco J. Pérez-Reche,
Sergei N. Taraskin, Wilfred Otten, Douglas J. Bailey, and Christopher A. Gilligan

# Text S1: Epidemic processes and percolation

## SIR processes

We consider a stochastic SIR process on a lattice, where a pathogen is transmitted between nearest neighbours. Within this model, a susceptible (S) host that is reached by the pathogen at some time $t_0 = 0$ (set to 0 for convenience) switches to the infectious (I) state, and can in turn transmit the pathogen to its susceptible neighbours according to an inhomogeneous Poisson process with infection rate $\beta(t)$. The probability that the pathogen is ever transmitted from the infected site (*donor*) to the susceptible site (*recipient*) is given by the *transmissibility*:

$$\psi = 1 - \exp\left( - \int_0^\infty \beta(t)\, dt \right) . \tag{S1}$$

In many theoretical and practical cases, it is common to consider an *infectious period* $\tau$, after which the infectious site switches to the recovered or removed (R) state (hence, $\beta(t) = 0$ for $t > \tau$) and plays no role in the rest of the epidemic. More generally (as is the case for our experiment), the rate $\beta(t)$ can decay to 0 fast enough when $t \to \infty$ for the donor to be considered as *effectively* removed (not infectious any longer) for large times. The rate $\beta(t)$ (and thus the value of $\psi$) can be the same for all the hosts in the system (*homogeneous* system), or in general depend on the particular donor and recipient sites (*heterogeneous* system).

The key problem is whether or not an SIR epidemic, starting from a single infected site (as in our case, or from a small set of infected sites) will *invade* the system, i.e., infect a significant part of the population [1]. Since we are considering a stochastic process, it is convenient to define a *probability of invasion* $P_{\mathrm{inv}}$ for the epidemic, which will depend in general on $\psi$. In an infinite system ($N \to \infty$), where the epidemic can go on forever, invasion corresponds to the infection of an arbitrarily large number of hosts. In a finite system, the epidemic will stop spreading after a finite time, and the choice of a condition for invasion is not unique: it is based in general on the boundary conditions of the system (in our experiment, a triangular lattice with the boundaries arranged as the edges of a hexagon) and depends on the characteristics of the final patch of infected sites (in our case, the number of edges of the hexagon reached).

| SIR | Bond percolation |
|---|---|
| $\psi$ (transmissibility) | $p$ (bond probability) |
| $P_{\text{inv}}$ (probability of invasion) | $P_\infty$ (mass of the infinite cluster) |
| value of $\psi$ at the invasion threshold | $p_c^{\text{bond}}$ (bond percolation threshold) |
| $\psi \leq p_c^{\text{bond}}$ (non-invasive regime) | $p \leq p_c^{\text{bond}}$ (subcritical regime) |
| $\psi > p_c^{\text{bond}}$ (invasive regime) | $p > p_c^{\text{bond}}$ (supercritical regime) |

**Table S2**: **SIR processes and bond percolation: mapping.** Parameters and regimes of a homogeneous SIR process (left column) are in the same row with the corresponding parameters and regimes from bond percolation (right).

## Percolation and epidemic invasion

In the bond percolation problem [2], each bond of a lattice is "open" with probability $p$ and "closed" with probability $1-p$. Sites connected by open bonds form clusters, which increase in size and number as $p$ increases from 0. In an infinite system, an *infinite cluster* appears when $p$ is greater than a critical value $p_c^{\text{bond}}$ (the bond percolation threshold). The value of $p_c^{\text{bond}}$ depends on the topology of the lattice, e.g., $p_c^{\text{bond}} = 0.5$ for a square lattice and $p_c^{\text{bond}} \simeq 0.347$ for a triangular lattice [2]. The equivalent of the infinite cluster in finite systems is the *spanning cluster*, which connects opposite boundaries of the lattice. The probability that a randomly chosen site belongs to the infinite cluster, $P_\infty(p)$ (relative "mass" of the infinite cluster), is 0 below the percolation threshold (subcritical regime) and grows continuously from $P_\infty(p_c^{\text{bond}}) = 0$ to $P_\infty(1) = 1$ above the percolation threshold (supercritical regime).

A rigorous link with the SIR epidemic model can be made [3, 4] by interpreting the "open" ("closed") state of a bond as a successful (unsuccessful) event of pathogen transmission along the bond. The bond probability $p$ is thus identified with the transmissibility $\psi$. The spread of the SIR epidemic is then equivalent to a process where open bonds are sequentially joined to infected sites (with probability $\psi$), thereby infecting the new attached sites, and a cluster of sites of the bond-percolation problem is grown from the initial infected site. The invasion threshold of the system, corresponding to the occurrence of an invasive patch of disease (equivalent to an infinite cluster of sites), is then given by the condition $\psi = p_c^{\text{bond}}$, and the probability of invasion, $P_{\text{inv}}$, is equal to the mass of the infinite cluster $P_\infty$. The critical value for the transmissibility $\psi = p_c^{\text{bond}}$ separates a *non-invasive regime* for $\psi \leq p_c^{\text{bond}}$ (where $P_{\text{inv}}(\psi) = 0$, i.e., the epidemic will never invade) from an *invasive regime* for $\psi > p_c^{\text{bond}}$ (where $P_{\text{inv}}(\psi) > 0$ and the epidemic has a non-zero probability to invade). The described mapping is summarised in Table S2.
In an alternative formulation, that has been used to study the connectivity of habitable sites in the landscape [5, 6], sites (instead of bonds) are occupied with probability $p$, and a percolating cluster of adjacent sites occurs at a value $p_c^{\text{site}}$ (site percolation threshold; e.g., $p_c^{\text{site}} \simeq 0.593$ for a square lattice and $p_c^{\text{site}} = 0.5$ for a triangular lattice [2]).

The theory for homogeneous systems was experimentally validated [5, 7] studying the spread of the fungal pathogen and saprotroph *R. solani* in lattice populations of nutrient sites in Petri plates. The fungal colonization process was shown to be experimentally equivalent to (and properly described by) SIR epidemic spread: here, the transmissibility $\psi$ represents the probability of colonisation between neighbouring nutrient sites, and a host going through the susceptible, infected, and removed stages is replaced by a nutrient site being successively uncolonised, colonised by the fungus, and depleted of nutrient [7, 8]. Microcosm experiments [7] showed the existence of thresholds for fungal invasion on a triangular lattice of nutrient sites, corresponding to a value of $\psi$ close to the predicted value $p_c^{\mathrm{bond}}$.

## Invasion in heterogeneous systems

In [9], we considered SIR epidemic spread in a *heterogeneous* population, where $\psi$ is a random number drawn for each donor site from a probability distribution. In this case, a simple mapping such as in the homogeneous case is not possible anymore, and only a few analytical results are available. If one considers the dependence of $P_{\mathrm{inv}}$ on $\langle\psi\rangle_{\mathrm{pop}}$ (the average of $\psi$ over all the sites in the population), it has previously been shown [3, 10] that the critical value of $\langle\psi\rangle_{\mathrm{pop}}$, separating the invasive and non-invasive regimes, lies in general between the bond- and site-percolation thresholds:

$$p_c^{\mathrm{bond}} \leq \langle\psi\rangle_{\mathrm{pop}} \leq p_c^{\mathrm{site}} \ . \tag{S2}$$

These results were extended with numerical methods, by considering explicitly the variance of the distribution for $\psi$, $\sigma^2_{\mathrm{pop}}$, for several classes of models [9]. We restrict here to the case of a two-host system, where hosts belonging to two different classes, A and B (with respective transmissibilities $\psi_A$ and $\psi_B$) occupy a fraction $\rho_A$ and $\rho_B = 1 - \rho_A$, respectively, of the lattice sites. The mean and variance of $\psi$ in this case are:

$$\langle\psi\rangle_{\mathrm{pop}} = \rho_A\psi_A + (1-\rho_A)\psi_B \tag{S3a}$$

$$\sigma^2_{\mathrm{pop}} \quad = \rho_A(1-\rho_A)(\psi_A-\psi_B)^2 \ , \tag{S3b}$$

so that the system is homogeneous ($\sigma^2_{\mathrm{pop}} = 0$) when one of the following conditions is satisfied: $\psi_A = \psi_B$; $\rho_A = 0$; $\rho_A = 1$.

The system used in our experiment is described by a particular case of Equations (S3): sites of type A are filled with nutrient ($\psi_A \equiv \psi_{\mathrm{site}}$, $\rho_A \equiv \rho$) and sites of type B are left empty ($\psi_B = 0$), which yields the expressions in manuscript Equation 1:

$$\langle\psi\rangle_{\mathrm{pop}} = \rho\,\psi_{\mathrm{site}}$$

$$\sigma^2_{\mathrm{pop}} = \rho\,(1-\rho)\,\psi^2_{\mathrm{site}} \ .$$

We use $\sigma^2_{\text{pop}}$ to quantify the degree of heterogeneity of the system.
For the preliminary simulations in the present paper (manuscript Figure 1) and in [9], we used a method based on numerical simulations in order to find (i) the dependence of $P_{\text{inv}}$ on $\langle\psi\rangle_{\text{pop}}$ and $\sigma^2_{\text{pop}}$ for several different system sizes, and (ii) the phase boundary for invasion. A brief description follows.

Given a system of size (number of sites) $N$, and a value of $\rho$ and $\psi_{\text{site}}$ from manuscript Equation 1, a *realisation* of heterogeneity corresponds to a particular, randomly generated configuration of "filled" and empty sites. For a given combination of $\rho$ and $\psi_{\text{site}}$ (corresponding to a combination of $\langle\psi\rangle_{\text{pop}}$ and $\sigma^2_{\text{pop}}$), several realisations ($10^2 - 10^3$) of heterogeneity were generated; for each realisation, the epidemic process was run once, starting from a single initial infection, and recording whether invasion had occurred or not when the process ended. The fraction of realisations where invasion had occurred was taken as the probability of invasion $P_{\text{inv}}$ for that particular combination of $\langle\psi\rangle_{\text{pop}}$ and $\sigma^2_{\text{pop}}$.

The phase boundary, and invasion threshold in general, are only uniquely and rigorously defined in the limit $N \to \infty$, and they have to be inferred from finite-size simulations. The phase boundary in manuscript Figure 3 (dash-dotted line) was found using finite size scaling and data collapse [2, 9], and extrapolating the invasion threshold for the system from curves for $P_{\text{inv}}$ obtained for different system sizes. The phase boundary of the "large" ($N = 24031$) system in manuscript Figure 1 was found using a cutoff method: the phase boundary was defined as the set of points where $P_{\text{inv}} = 0.2$ (we have checked that setting the threshold to other small values around $P_{\text{inv}} = 0.2$ give similar results). The resulting phase diagram is close to that obtained withmanuscript e scaling and shown in manuscript Figure 3.

The main result, already discussed in the manuscript Introduction, is that the probability of epidemic invasion $P_{\text{inv}}$ increases monotonically with $\langle\psi\rangle_{\text{pop}}$ (for a given $\sigma^2_{\text{pop}}$), but decreases monotonically with $\sigma^2_{\text{pop}}$ (for a given $\langle\psi\rangle_{\text{pop}}$) [9] (manuscript Figure 1). For a sufficiently large system (manuscript Figure 1A), the epidemic exhibits a "threshold" behaviour, with $P_{\text{inv}}$ dropping suddenly from a positive value (*invasive* region in the parameter space $\big(\langle\psi\rangle_{\text{pop}}, \sigma^2_{\text{pop}}\big)$) to zero (*non-invasive* region). Such behaviour is summarised by the phase diagram in manuscript Figure 1A, where the invasive and non-invasive regions in the parameter space are separated by a phase boundary (solid line in manuscript Figure 1B, corresponding to the steep transition in manuscript Figure 1A). In the phase diagram, three relevant intervals for the mean transmissibility can be identified. Systems with $\langle\psi\rangle_{\text{pop}} < p_c^{\text{bond}}$ or $\langle\psi\rangle_{\text{pop}} > p_c^{\text{site}}$ are respectively non-invasive and invasive, independent of the value of $\sigma^2_{\text{pop}}$. However, for $p_c^{\text{bond}} < \langle\psi\rangle_{\text{pop}} < p_c^{\text{site}}$, invasion also depends on $\sigma^2_{\text{pop}}$. Notably, in this interval it is possible to go from the invasive to the non-invasive regime and *vice-versa* by keeping $\langle\psi\rangle_{\text{pop}}$ constant and changing only $\sigma^2_{\text{pop}}$ (e.g., along the dash-dotted line in manuscript Figure 1B). In relatively small systems (manuscript Figure 1C), the transition is "smeared out" and the sharp phase boundary disappears, but it is still possible to identify regions with low and high probability of invasion, and "paths" with constant $\langle\psi\rangle_{\text{pop}}$ along which $P_{\text{inv}}$ decreases with $\sigma^2_{\text{pop}}$ (e.g., the path along the dash-dotted line in manuscript

Figure 1D).

It was also shown [9] that different families of distributions for $\psi$ yielded very similar phase boundaries. Hence, the first two moments of the distribution for $\psi$, $\langle\psi\rangle_{\mathrm{pop}}$ and $\sigma^2_{\mathrm{pop}}$, can determine the position of the boundary with good accuracy (at least for lattices with low coordination numbers [9]). In general, quadratic functions in $\langle\psi\rangle_{\mathrm{pop}}$ and $\sigma^2_{\mathrm{pop}}$ were found to fit the numerical data very well (for different lattices), but an analytical expression for the phase boundary could not be found, nor for the dependence of $P_{\mathrm{inv}}$ on $\langle\psi\rangle_{\mathrm{pop}}$ and $\sigma^2_{\mathrm{pop}}$.

Recently, disease spread with individual heterogeneity has been modeled for complex networks systems [11–15]. In common with the results for regular lattices [3, 9], network models predict that $P_{\mathrm{inv}}$ is maximised when transmission of infection is homogeneous, and minimised when the variance in transmission is maximal [11–13]. On the other hand (and differently than for lattices), network models predict that heterogeneity does not affect the threshold for invasion for the system, which has the same value as for the corresponding homogeneous system [11–13]. The reason for the difference is in the network topology: complex networks are *locally tree-like* (short cycles are rare and can be neglected): for a tree graph, the bond- and site-percolation threshold coincide, $p_c^{\mathrm{bond}} = p_c^{\mathrm{site}}$, and the interval in Equation S2 for the value of $\langle\psi\rangle_{\mathrm{pop}}$ degenerates into a single point. In the present paper, the analysis is restricted to regular lattices, where the contact structure –the distribution of host connectivity– is homogeneous by definition, since the number of neighbours of each site is constant. Such structure is appropriate for a system of sessile or very locally mobile hosts (e.g., for a plant population or hosts in a fixed habitat), and when the connectivity of the hosts does not change significantly during the spread of the epidemic. By allowing variability in contact structure, it is possible to model disease spread in cases when the connectivity of individuals can vary greatly across the population (typically, human and animal diseases) [16]: such has been the case for network-based metapopulation models [17–20] and agent-based models [21] for disease spread.

We also remark that in our heterogeneous model, as in the experiment described in the present paper, the transmissibility $\psi$ depends only on the *donor* site (hence, it is related to the *infectiousness* of the site). Recent models, both on complex networks [12, 14, 15, 22] and on lattices [22, 23], have taken into account the case where $\psi$ also depends on the *recipient* site (hence, it is also *susceptibility*-related). A nice analytical result is the proof that, under quite general assumptions, the bounds in Equation S2 still hold [22].

# References


[1] Diekmann O, Heesterbeek JAP (2000) Mathematical Epidemiology of Infectious Diseases: Model Building, Analysis, and Interpretation. John Wiley.

[2] Stauffer D, Aharony A (1991) Introduction to Percolation Theory. London: Taylor and Francis.

[3] Kuulasmaa K (1982) The spatial general epidemic and locally dependent random graphs. J Appl Prob 19: 745–758.

[4] Grassberger P (1983) On the critical behavior of the general epidemic process and dynamical percolation. Math Biosci 63: 157-172.

[5] Otten W, Bailey DJ, Gilligan CA (2004) Empirical evidence of spatial thresholds to control invasion of fungal parasites and saprotrophs. New Phytol 163: 125-132.

[6] Davis S, Trapman P, Leirs H, Begon M, Heesterbeek J (2008) The abundance threshold for plague as a critical percolation phenomenon. Nature 454: 634-637.

[7] Bailey DJ, Otten W, Gilligan CA (2000) Saprotrophic invasion by the soil-borne fungal plant pathogen *rhizoctonia solani* and percolation thresholds. New Phytol 146: 535-544.

[8] Bailey DJ, Gilligan CA (1997) Components of pathozone behaviour. New Phytol 135: 475-490.

[9] Neri FM, Pérez-Reche FJ, Taraskin SN, Gilligan CA (2011) Heterogeneity in SIR epidemics on lattices. J R Soc Interface 8: 201–209.

[10] Cox JT, Durrett R (1988) Limit theorems for the spread of epidemics and forest fires. Stoch Proc Appl 30: 171–191.

[11] Kenah E, Robins JM (2007) Second look at the spread of epidemics on networks. Phys Rev E 76: 036113.

[12] Miller JC (2007) Epidemic size and probability in populations with heterogeneous infectivity and susceptibility. Phys Rev E 76: 010101(R).

[13] Newman MEJ (2002) Spread of epidemic disease on networks. Phys Rev E 66: 016128.

[14] Miller JC (2009) Percolation and epidemics in random clustered networks. Phys Rev E 80: 020901(R).

[15] Miller JC (2009) Spread of infectious disease through clustered populations. J R Soc Interface 6: 1121–1134.

[16] Bansal S, Grenfell BT, Meyers LA (2007) When individual behaviour matters: homogeneous and network models in epidemiology. J R Soc Interface 4: 879–891.

[17] Colizza V, Vespignani A (2007) Invasion threshold in heterogeneous metapopulation networks. Phys Rev Lett 99: 148701.

[18] Colizza V, Barrat A, Barthélemy M, Vespignani A (2007) Epidemic predictability in meta-population models with heterogeneous couplings: the impact of disease parameter values. International Journal of Bifurcation and Chaos 17: 2491-2500.

[19] Colizza V, Pastor-Satorras R, Vespignani A (2007) Reactiondiffusion processes and metapopulation models in heterogeneous networks. Nature Physics 3: 276–282.

[20] Balcan D, Colizza V, Goncalves B, Hu H, Ramasco JJ, et al. (2009) Multiscale mobility networks and the spatial spreading of infectious diseases. Proc Natl Acad Sci USA 106: 21484-21489.

[21] Eubank S, Guclu H, Kumar VSA, Marathe MV, Srinivasan A, et al. (2004) Modelling disease outbreaks in realistic urban social networks. Nature 429: 180–184.

[22] Miller JC (2008) Bounding the size and probability of epidemics on networks. J Appl Probab 45(2): 498–512.

[23] Meester R, Trapman P (2008) Bounding basic characteristics of spatial epidemics with a new percolation model. preprint at arXiv:08124353v2 .
