# Supplementary material for: The Effect of Heterogeneity on Invasion in Spatial Epidemics: From Theory to Experimental Evidence in a Model System
Source: PLoS Comput Biol. 2011 Sep 29;7(9):e1002174. doi: 10.1371/journal.pcbi.1002174 (PMC3182855; doi:10.1371/journal.pcbi.1002174)
Supplement: Text S2 — We describe the colonisation experiments performed to find the value of the transmissibility as a function of the nutrient concentration. (PDF) [file pcbi.1002174.s003.pdf]

The effect of heterogeneity on invasion in spatial epidemics: from theory to experimental evidence in a model system

Franco M. Neri, Anne Bates, Winnie S. Füchtbauer, Francisco J. Pérez-Reche,
Sergei N. Taraskin, Wilfred Otten, Douglas J. Bailey, and Christopher A. Gilligan

Text S2: Quantifying rates and probabilities of transmission in colonisation experiments.

Colonisation experiments [1,2] were performed before the population experiment to find the value of ψ_{site} as a function of the nutrient concentration. We used pairs of agar dots (potato dextrose agar, 20 μL, 3 mm diameter), with seven different nutrient concentrations ranging from 0.5% to 12%. The pairs, comprising a donor and a recipient, were placed at 8 mm apart (from centre to centre) in Petri plates (90-mm diameter). There were 25 replicates of each concentration in a fully randomized design. The donor site of each agar pair was inoculated with a hyphal strand removed from the edge of a 4d-old colony of *R. solani* R5 (AG 2-1) grown on water agar. We placed moist filter paper in the lid of each Petri plate to avoid desiccation of the agar. The plates were sealed and incubated in the dark at 23°C. Recipient dots were assessed daily for colonisation for 21 days. For each nutrient concentration, the fraction of colonised recipients was recorded as a function of time.

Figure S1 shows the results of the colonisation experiments. The probability of colonisation as a function of time (solid curves in Figure S1A) was fitted to the fraction of colonised recipients using manuscript Equation 2:

$$\begin{aligned} f_W(t) &= \psi_{\text{site}} \left(t^{k-1}/\lambda^k\right) e^{-(t/\lambda)^k} \\ F_W(t) &= \psi_{\text{site}}(1 - e^{-(t/\lambda)^k}), \end{aligned}$$

The probability ψ_{site} coincides with the asymptotic value of $F_W(t)$. The estimated values of ψ_{site} (Figure S1B) increase with the nutrient concentration, reaching the maximum transmissibility $\psi_{\text{site}} \simeq 1$ for concentrations 10% and 12%.

For the notional experimental treatments (Table S1), ψ_{site} and ρ were first found using manuscript Equation 1 (given the constraints $\langle\psi\rangle_{\text{pop}} \simeq 0.5$, σ^2_{pop} changing by ~ 0.05 between treatments), and the nutrient concentration was then calculated from ψ_{site} based on Figure S1B. Three out of six treatments (B, C, D) required concentrations in the interval between 3% and 5%. Since there were no experimental data in-between, we used a linear interpolation (Figure S1B) to infer the missing values.

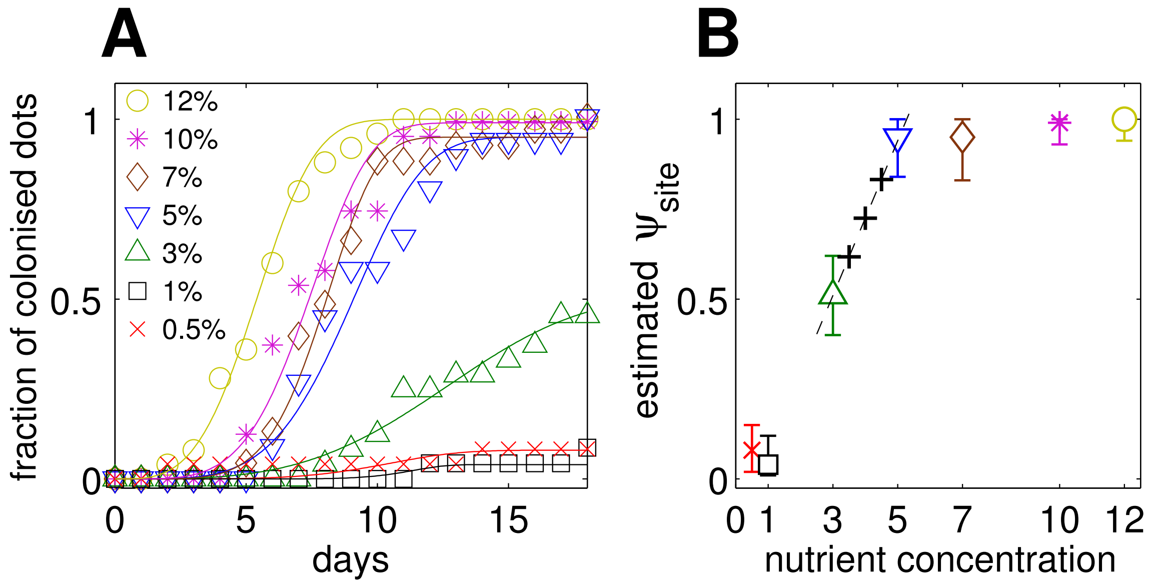

Figure S1: **Colonisation between isolated donor-recipient pairs for different nutrient concentrations.** (A) Colonisation dynamics: the fraction of replicated recipient sites that are colonised as a function of time for each concentration. The continuous lines are fitted Weibull curves (manuscript Equation 2). (B) The values of ψ_{site} estimated for each concentration, with error bars corresponding to the 95% confidence interval. The dotted line shows the linear interpolation between concentrations 3% and 5%, used to find values of ψ_{site} between 0.5 and 0.9 (indicated by crosses on the dotted line).

References

[1] Otten W, Bailey DJ, Gilligan CA (2004) Empirical evidence of spatial thresholds to control invasion of fungal parasites and saprotrophs. New Phytol 163: 125-132.

[2] Bailey DJ, Otten W, Gilligan CA (2000) Saprotrophic invasion by the soil-borne fungal plant pathogen *rhizoctonia solani* and percolation thresholds. New Phytol 146: 535-544.
