# Supplementary material for: The Effect of Heterogeneity on Invasion in Spatial Epidemics: From Theory to Experimental Evidence in a Model System
Source: PLoS Comput Biol. 2011 Sep 29;7(9):e1002174. doi: 10.1371/journal.pcbi.1002174 (PMC3182855; doi:10.1371/journal.pcbi.1002174)
Supplement: Text S3 — We give more details on the MCMC methods used for parameter estimation. We also provide a complete survey of the results of the estimation, and show how heterogeneity in transmissibility can be described by a simple hierarchical model. (PDF) [file pcbi.1002174.s004.pdf]

The effect of heterogeneity on invasion in spatial epidemics: from theory to experimental evidence in a model system

Franco M. Neri, Anne Bates, Winnie S. Füchtbauer, Francisco J. Pérez-Reche,
Sergei N. Taraskin, Wilfred Otten, Douglas J. Bailey, and Christopher A. Gilligan

Text S3: Parameter estimation and data analysis

Markov-chain Monte Carlo method

We fitted to the data a five-parameter model, and modelled the transmission of the fungal colony between two neighbouring sites as a time-inhomogeneous Poisson process [1], with waiting time distribution given by manuscript Equation 2, and two different values for the rate λ. The vector representing the parameters is $\boldsymbol{\theta} = (\psi_{\text{site}}, \lambda_1, \lambda_2, k, t_{\text{sw}})$, where ψ_{site} is the usual site transmissibility, k is a shape parameter, t_{sw} is the "switching" time for the transition from the slower initial process to the faster process, $\lambda = \lambda_1$ for $t \leq t_{\text{sw}}$, and $\lambda = \lambda_2$ for $t > t_{\text{sw}}$. Given the prior distribution of the parameters, $\pi(\boldsymbol{\theta})$, and the observed data D, we are interested in finding $\pi(\boldsymbol{\theta}|D)$, the posterior distribution of $\boldsymbol{\theta}$ given the data. According to Bayes' formula, $\pi(\boldsymbol{\theta}|D) \propto L(\boldsymbol{\theta})\pi(\boldsymbol{\theta})$, where the likelihood $L(\boldsymbol{\theta}) = \Pr(D|\boldsymbol{\theta})$ is given by the probability of the data conditioned on the parameters.

Assuming that we know exactly the time of colonisation t_j of each site j, the likelihood can be calculated explicitly as the product of the contributions from all those individual sites that are either infected (with probability density function $g_j(t_j)$) or not infected (with probability h_j) at the end of the experiment, $t_{\text{end}} = 41$ days:

$$L(\boldsymbol{\theta}) = \prod_{j \text{ infected}} g_j(t_j) \prod_{j \text{ not infected}} h_j, \tag{S4}$$

where:

$$g_j(t_j) = \phi_j(t_j) \ \exp\left(-\int_{t_i}^{t_j} \phi_j(t)dt\right) \tag{S5a}$$

$$h_j = \exp\left(-\int_{t_i}^{t_{\text{end}}} \phi_j(t)dt\right) . \tag{S5b}$$

Here, the *hazard function* for site j is given by $\phi_j(t) = \sum_i \beta_W(t - t_i)$, the sum being performed on all the potential donors of j (i.e., all the neighbouring sites i that are infected before j if j is infected, or infected before t_{end} if j is not infected). The nearest-neighbour infection rate $\beta_W(t)$ can be found analytically (not shown here) from manuscript Equation 2, adding the two-stage time dependence of the parameter λ, and using the relation $\beta_W(t) = f_W(t)/(1 - F_W(t))$ [1]. In our case, the exact colonisation times are unobserved (*censored*), because the status of each site

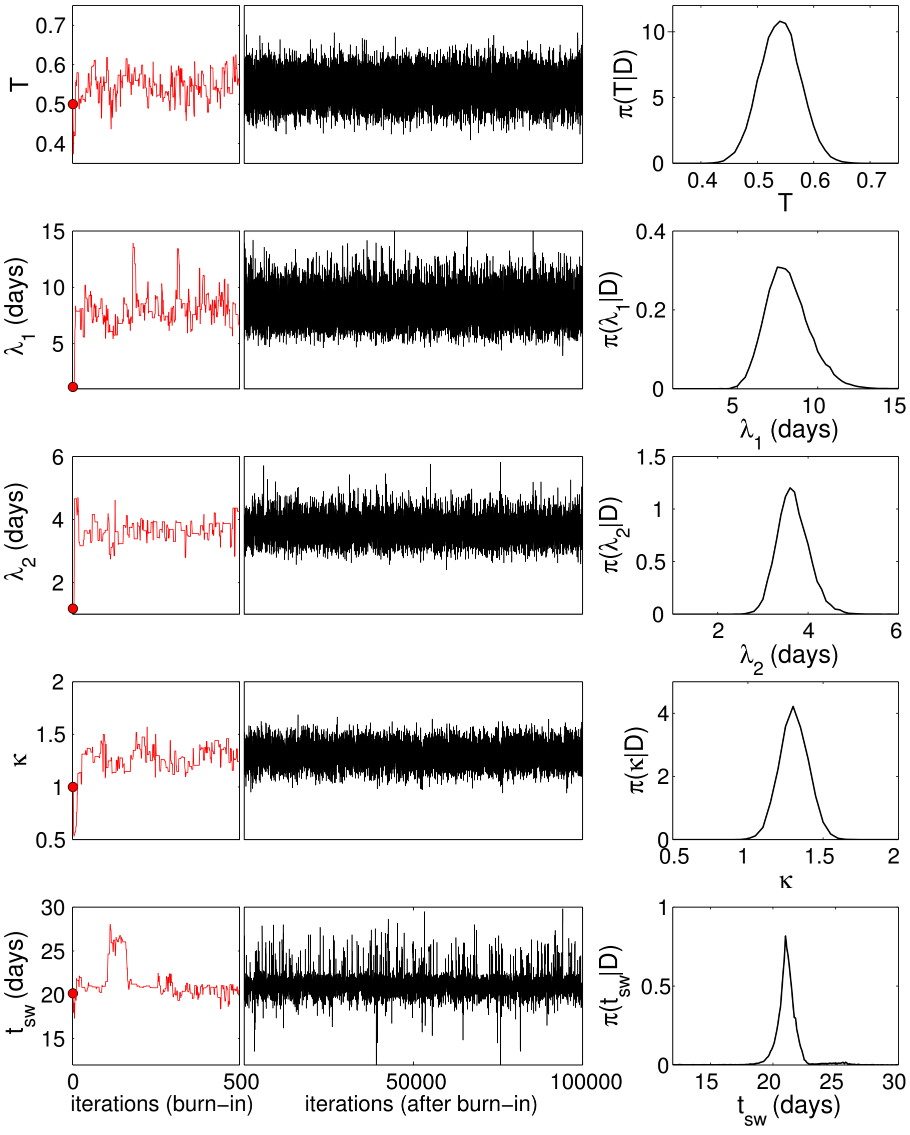

Figure S2: **Parameter estimation for one of the replicates of treatment C.** The replicate considered here is the same represented in magenta in manuscript Figure 5. For each of the five parameters of the model we plot: *(left)* the traceplot of the first 500 (burn-in) iterations; *(middle)* the complete traceplot; *(right)* the estimated posterior distribution.

is only observed at discrete (2-day or weekly) intervals. All the information we have about time t_j is that it falls within the window $(t_j^{\text{low}},\, t_j^{\text{up}})$, where t_j^{up} is the day when colonisation of j was first observed, and t_j^{low} the day of the previous survey. The likelihood has to be calculated from Equations (S4,S5) by integrating out the unobserved colonisation times. Such high-dimensional integrals are usually unfeasible in practice, and it is common to use numerical methods to obtain samples from the posterior distribution $\pi(\boldsymbol{\theta}|D)$.

In order to sample from $\pi(\boldsymbol{\theta}|D)$, we used an MCMC method with a Metropolis-Hastings algorithm [2]. The unobserved colonisation times $\{t_j\}$ were accounted for using data augmentation [3], i.e., treating them as parameters to be estimated and expanding the parameter vector: $\boldsymbol{\theta} \to (\boldsymbol{\theta},\, \{t_j\})$. However, additional care had to be taken for these parameters, since at each Monte Carlo step a pathway of transmission must exist between the site inoculated at time $t = 0$ and all the other colonised sites in the system (see discussions in [3, 4]).

We assumed independent priors for all the parameters: $\pi(\boldsymbol{\theta}) = \pi(T)\pi(\lambda_1)\pi(\lambda_2)\pi(k)\pi(t_{\text{sw}})$. Exponential priors were used for the parameters of the Weibull distribution: $\pi(\lambda_1) = \pi(\lambda_2) = \pi(k) = \text{Exp}(5)$. For ψ and t_{sw}, noninformative flat priors were used: $\pi(\psi) = U(0,\, 1)$, $\pi(t_{\text{sw}}) = U(0,\, t_{\text{end}})$. A uniform prior distribution was also used for each augmented colonisation time, but the support of this distribution in general could change in order to keep the pathway of transmission [4]. Every chain was run for 10^5 MCMC steps, discarding an initial burn-in period of $10^2 - 10^3$ steps. Assessment of the traceplots for each replicate (see Figure S2 for an example) shows convergence and good mixing of the Markov chains.

Results of parameter estimation

The posterior distributions for $\psi_{\text{site}}(T,\, r)$ for each treatment T and replicate r were used to calculate the new estimates $\widehat{\psi}_{\text{site}}(T,\, r)$, $\langle\widehat{\psi}\rangle_{\text{pop}}(T,\, r)$ and $\widehat{\sigma^2}_{\text{pop}}(T,\, r)$ for each population; an example is given in manuscript Figure 5. In Figure S3, we show the complete set of results, with the new estimates for the parameters grouped by treatments and compared with their original nominal values. Figure S4 shows the corresponding histograms for each treatment. The average and standard deviation of the new estimates over each treatment are shown in Table S1.

From Figures S3 and S4, two main results emerge. First, there is a systematic shift of ψ_{site} (consequently, of $\langle\psi\rangle_{\text{pop}}$ and σ^2_{pop}) to the left of the notional value, starting with treatment D and increasing for treatments E and F (scatter plots vs. horizontal lines in the panels of Figure S3; histograms vs. circles in the panels of Figure S4). The discrepancy between nominal (pair-experiment) and estimated (population-experiment) values of the transmissibility thus becomes more significant with increasingly high nutrient concentrations. This result can be interpreted by stating that the transmissibility within a population tends to "saturate" for high nutrient concentrations (for reasons still not known), differently than in pair experiments. The second, more important effect is a strong within-treatment variability, already discussed in the manuscript and summarized in Table S1. The effect is evident, for example, in the histograms for treatment

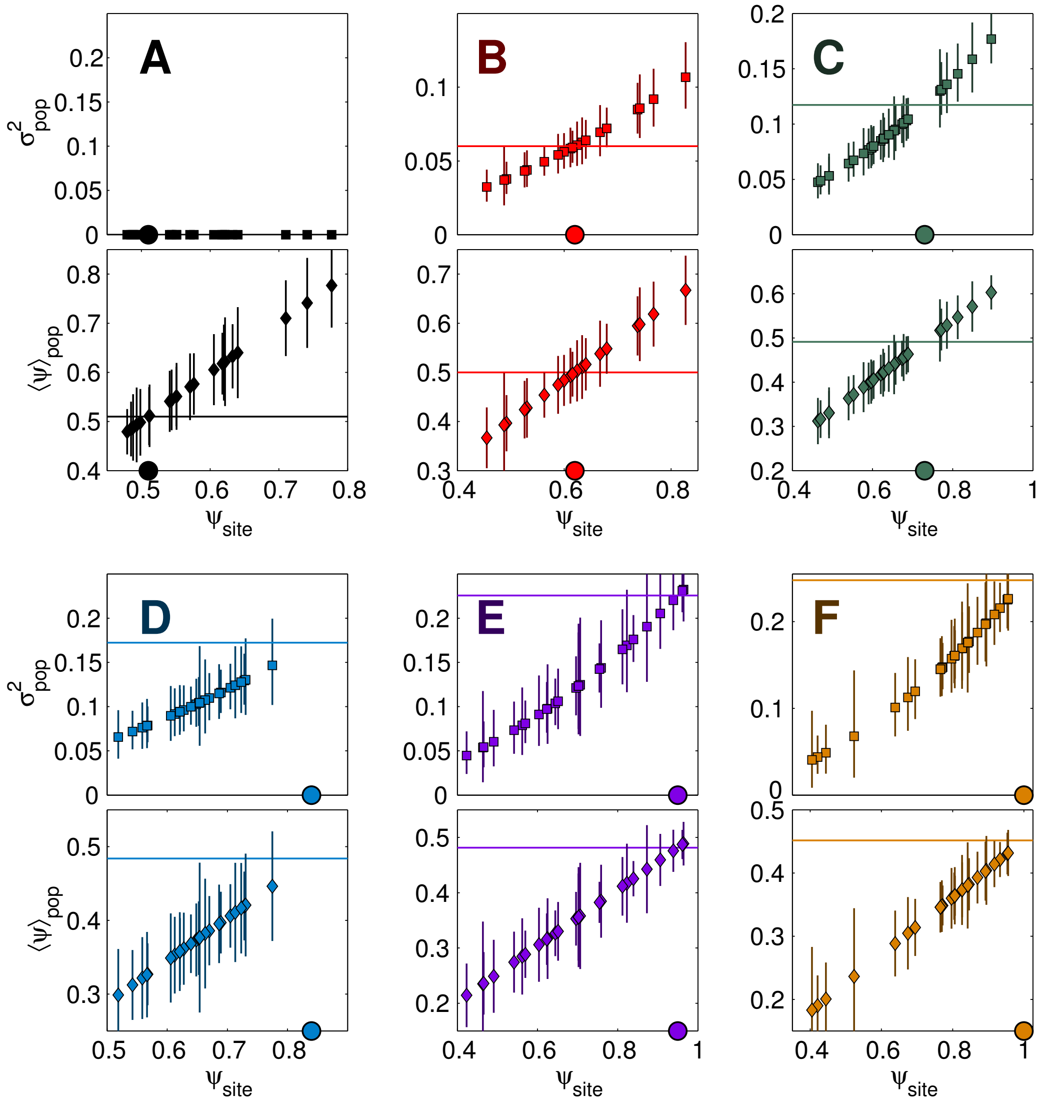

Figure S3: **Within-treatment variation of the transmissibility.** Estimated values of ψ_{site}, $\langle\psi\rangle_{\mathrm{pop}}$, σ^2_{pop}, grouped by treatment. For each treatment T, the new individual-replicate estimates of $\langle\psi\rangle_{\mathrm{pop}}(T,\, r)$ (coloured symbols) are plotted in the lower panel as a function of the corresponding estimates of $\psi_{\mathrm{site}}(T,\, r)$, with error bars corresponding to the 95% credible interval (error bars for $\widehat{\psi}_{\mathrm{site}}(T,\, r)$ are not shown). The new estimates of $\sigma^2_{\mathrm{pop}}(T,\, r)$ are plotted in the same way in the upper panel. The coloured circles on the ψ_{site} axis mark the nominal value of ψ_{site}; the coloured horizontal lines in the lower and upper panel mark the the nominal value of $\langle\psi\rangle_{\mathrm{pop}}$ and σ^2_{pop}, respectively.

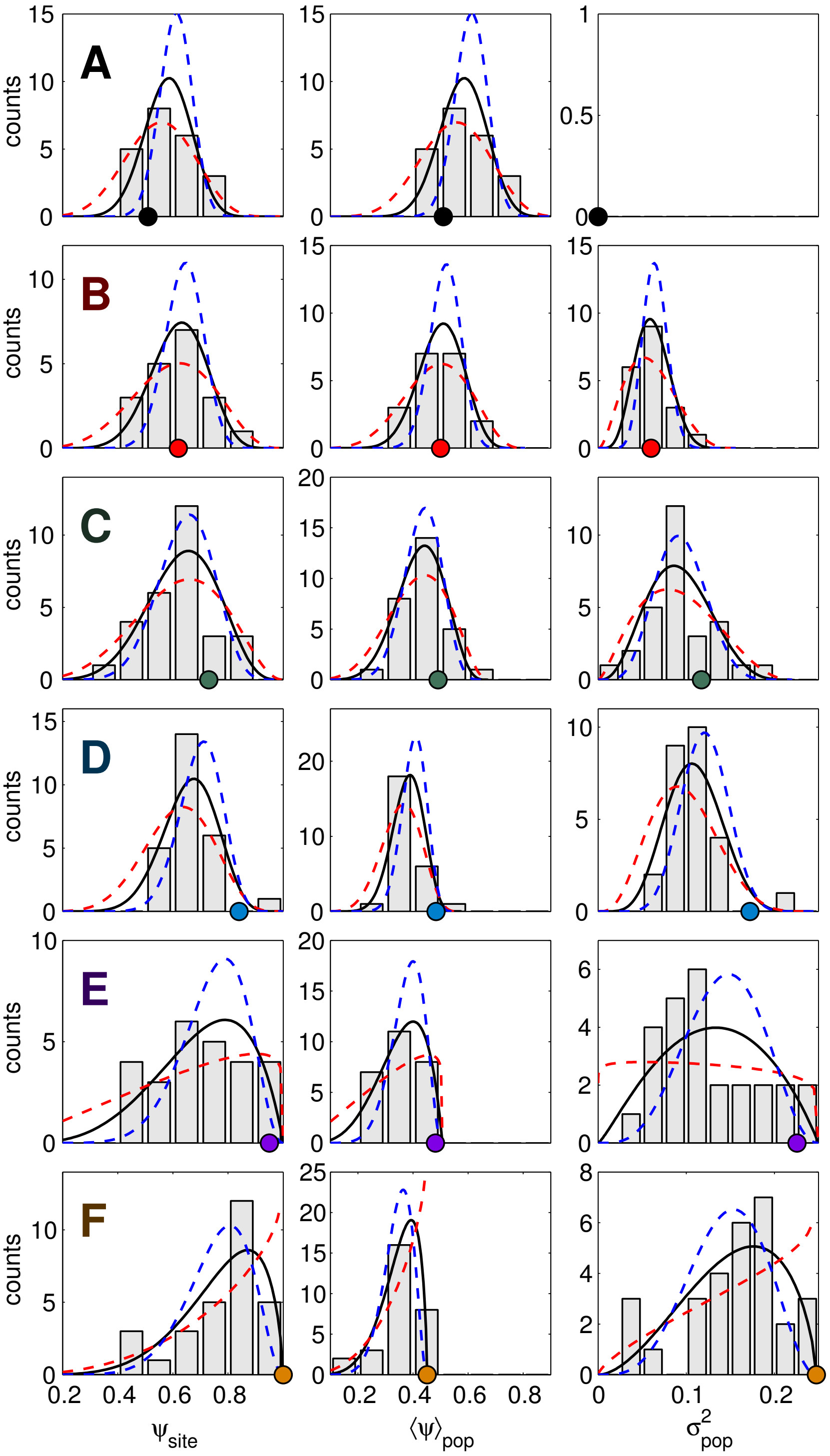

Figure S4: **Distribution of within-treatment heterogeneity.** Histograms of the new estimates $\widehat{\psi}_{\rm site}(T,\,r)$, $\langle\widehat{\psi}\rangle_{\rm pop}(T,\,r)$ and $\widehat{\sigma^2}_{\rm pop}(T,\,r)$ from Figure S3. Coloured circles on the horizontal axes mark the nominal value of the corresponding parameter. Beta distributions (Equation S6) fitted to the distributions for $\widehat{\psi}_{\rm site}(T,\,r)$ are shown overlayed to the histograms (rescaled for display): best fit (solid black curve) and 95% confidence intervals (dashed red and blue curves). The distributions for $\langle\widehat{\psi}\rangle_{\rm pop}(T,\,r)$ and $\widehat{\sigma^2}_{\rm pop}(T,\,r)$, calculated analytically from those for $\widehat{\psi}_{\rm site}(T,\,r)$ (best fit and 95% confidence interval), are also overlayed to the corresponding histograms.

F (Figure S4), where the empirical distribution for $\langle\widehat{\psi}\rangle_{\text{pop}}(T, r)$ spans the interval (0.1, 0.5), and the empirical distribution for $\widehat{\sigma^2}_{\text{pop}}(T, r)$ covers almost all the available interval (0, 0.25). As explained in the manuscript, the initial, nominal treatments were accordingly dropped in favour of a pooled analysis.

Fitting a model for heterogeneity

We show here how within-treatment heterogeneity in transmissibility can be modelled within a hierarchical Bayesian framework [2]. We assume that the value of the transmissibility $\psi_{\text{site}}(T, r)$ is drawn for each r from a prior distribution (a "hyperprior" [2]), and that the parameters of such distribution depend on T only. A Beta distribution was chosen as the hyperprior:

$$\text{Beta}(\psi_{\text{site}};\, a,\, b) = \frac{\Gamma(a+b)}{\Gamma(a)\Gamma(b)} \psi_{\text{site}}^{a-1} \psi_{\text{site}}^{b-1}, \tag{S6}$$

where Γ is the Euler Gamma function, and a, $b > 0$ depend on T.

The parameters of the distribution (S6) were estimated for each treatment T. We used an "empirical" Bayes method [5], and fitted the distribution to the individual estimates $\widehat{\psi}_{\text{site}}(T, r)$ (whereas a "fully Bayesian" method would involve including the hyperprior distribution from the start in the MCMC estimation [2]). Table S3 shows the estimated parameters a and b (best fit and 95% C.I.). The curves corresponding to the best-fit parameters (together with the curves corresponding to the 95% C.I. for a and b) are shown in Figure S4, overlayed to the histograms. The distributions for $\langle\widehat{\psi}\rangle_{\text{pop}}(T, r)$ and $\widehat{\sigma^2}_{\text{pop}}(T, r)$ (also shown in Figure S4) can be calculated analytically from the Beta distribution for $\widehat{\psi}_{\text{site}}(T, r)$ and from manuscript Equation 1 using standard methods [6]. The Beta distribution accounts for the asymmetry in the histograms for treatments E and F (Figure S4); the mode and absolute value of the best-fit distributions increase consistently from treatment A to treatment F (Table S3).

Treatment	a	b	mean	mode	std	skewness
A	**19.5** (8.85, 43.1)	**14.1** (7.2, 27.5)	0.58	0.59	0.08	−0.11
B	**14.2** (6.53, 31.1)	**8.69** (4.33, 17.5)	0.62	0.63	0.1	−0.2
C	**8.83** (5.39, 14.5)	**5.09** (3.27, 7.93)	0.63	0.66	0.12	−0.27
D	**15.2** (9.39, 24.5)	**7.78** (5.77, 10.5)	0.66	0.68	0.1	−0.27
E	**4.62** (2.1, 10.2)	**1.97** (1.12, 3.45)	0.70	0.79	0.17	−0.57
F	**5.87** (3.41, 10.1)	**1.72** (0.93, 3.18)	0.77	0.87	0.14	−0.8

Table S3: **Fitting a model for heterogeneity.** Parameters a and b of the Beta distribution (S6) used to model heterogeneity in $\psi_{\text{site}}(T, r)$ within each treatment. Best-fit parameters are in bold, 95% confidence intervals in parentheses. The curves corresponding to these parameters are plotted in panels of the left column of Figure S4. The mean, mode, standard deviation (std), and skewness values refer to the best-fit distribution.

References

[1] Cox DR, Isham V (1980) Point Processes, volume 12 of *Monographs on Applied Probability and Statistics*. London: Chapman and Hall.

[2] Gelman A, Carlin JB, Stern HS, Rubin DB (2003) Bayesian Data Analysis. New York: Chapman & Hall/CRC.

[3] Gibson GJ, Renshaw E (1998) Estimating parameters in stochastic compartmental models using markov chain methods. IMA J Math Appl Med 15: 19-40.

[4] Gibson GJ, Otten W, Filipe JAN, Cook AR, Marion G, et al. (2006) Bayesian estimation for percolation models of disease spread in plant populations. Stat Comput 16: 391-402.

[5] Carlin BP, Louis TA (2000) Bayes and Empirical Bayes Methods for Data Analysis. New York: Chapman & Hall/CRC.

[6] Rohatgi VK, Saleh AKME (2001) An Introduction to Probability and Statistics. New York: John Wiley & Sons.
